# Determination of the Critical Speed of a Cracked Shaft from Experimental Data

**DOI:** 10.3390/s22249777

**Published:** 2022-12-13

**Authors:** Belén Muñoz-Abella, Laura Montero, Patricia Rubio, Lourdes Rubio

**Affiliations:** Mechanical Engineering Department, University Carlos III of Madrid, 28911 Leganés, Spain

**Keywords:** critical speed, cracked shafts, rotating shafts, frequency spectrum

## Abstract

In this work, a procedure to obtain an accurate value of the critical speed of a cracked shaft is presented. The method is based on the transversal displacements of the cracked section when the shaft is rotating at submultiples of the critical speed. The SERR (Strain Energy Ralease Rate) theory and the CCL (Crack Closure Line) approach are used to analyse the proposed methodology for considering the behaviour of the crack. In order to obtain the best information and to define the procedure, the orbits and the frequency spectra at different subcritical rotational speed intervals are analyzed by means of the Fast Fourier Transform. The comparison of the maximum values of the FFT peaks within the intervals allows the subcritical speed to be determined, along with the value of the critical speed. When verified, the proposed procedure is applied to shafts with the same geometry and material and with cracks of increasing depth. The results show that the critical speed diminishes with the severity of the crack, as expected. A comparison is made between the critical speed obtained using the vertical and the horizontal displacements, finding no remarkable differences, meaning that in practical applications only one sensor for one of the displacements (in the vertical or horizontal direction) is needed to determine the critical speed. This is one of the main contributions of the paper, as it means that the orbits of the shaft are not needed. Finally, after this study we can conclude that the best results are achieved when the critical speed is obtained using data displacement in only one direction within the intervals around $\frac{1}{2}$ or $\frac{1}{3}$ of the critical speed.

## 1. Introduction

One of the main components of rotary machines are the rotating shafts. Examples include rotors, turbines, compressors, and more. Due to service conditions, these rotating components may present growing cracks that can lead to catastrophic failures, as mentioned in many works, previous i.e., [1]. Therefore, studies of the behavior of cracked rotary shafts are required.

Any kind of defect that appears in a mechanical element produces a change in its behavior. For example, the appearance of fatigue cracks causes a decrease in the component stiffness, which affects the static and dynamic behavior. This reduction of stiffness produces an increase of the displacements and a decrease of the natural frequencies. Numerous studies have focused on the analysis of the dynamic behavior of a cracked rotating shaft. Changes in the behavior of the mechanical elements have sometimes been used to detect and identify defects in the components by solving an inverse problem [2,3,4,5,6,7,8,9,10,11,12].

Regarding specific elements such as rotating shafts, the presence of cracks affects the orbits described by the different sections of the rotors as well as the critical speeds. The changes in the behavior of cracked shaft are summarized in the reviews of Wauer [13], Gasch [14], Dimarogonas [15], and Papadopoulos [16], among others.

Changes in displacement and vibration frequency can be taken as defect indicators. To this end, many authors have suggested using indicators of the nonlinear behavior of the cracked shaft, such as the natural frequency and the orbits, in order to detect the presence of cracks [17,18,19,20,21,22,23,24]. The reduction of the the critical speed turns out to be a very good indicator of the presence of cracks [25] in rotating shafts.

Different models have been used to study the dynamic behavior of cracked shafts, among which the most used is the Jeffcott Rotor model [14,16,26,27,28,29,30,31]. Although it is a simplification, it retains the basic characteristics of the dynamics of shafts, which allows an excellent understanding of the phenomena that occur in rotary shafts, including possible misaligment and imbalance.

On the other hand, both the shaft and the cracks have to be modeled. Several authors have taken the simplest model, that is, a crack that is always open [16,32], while others have considered a hinge model [26,33,34,35,36], as well as a more complicated version that opens and closes gradually during the rotation of the shaft, called a “breathing crack” [22,28,30,31,37,38]. Among the breathing crack models, the most widely used is that based on the Strain Energy Release Rate (SERR) and fracture mechanics concepts [27,28,30,31,38].

During the rotation of the shaft, each section along the length of the shaft describes what is called an orbit, which is the representation of the two main transversal displacements perpendicular to each other. The study of the orbits at certain speeds of rotation, such as the critical speed or its multiples and submultiples, provides very good information about the crack [20,22,39,40,41,42,43]. It is well known that the orbits of cracked shafts are elliptical when they perform out of critical or subcritical speeds. However, they lose their elliptical shape when passing through the critical or subcritical speeds, when a number of loops appear according to the speed of rotation ([20,44]). Frequently, speeds near $\frac{1}{4}$, $\frac{1}{3}$, $\frac{1}{2}$ of the critical speed are studied, as these are the speeds at which the orbits present the most significant changes [22]. If the orbit has *n* internal loops, the rotation speed is considered to be close to $\frac{1}{n+1}\omega_{c}$ ([22,39,40,41,42,43,45]). Therefore, the number of the loops in the orbits at a given rotation speed can help to identify the critical speed of the cracked shaft. However, because the size of the loops depends on the crack depth [20], swallow cracks may be undetectable using this procedure, and other methodologies should be used to detect the presence of cracks together with orbit evaluation, such as frequency spectrum analysis [46].

In the case of damaged shafts, the analysis of signals using the frequency spectra is a quick and effective way to see the differences in the behaviors of healthy and cracked shafts. The occurrence of certain frequencies and their relative amplitudes may indicate the type of defect the shaft presents [25,30,43,47,48]. The frequency spectrum of an unbalanced shaft shows a frequency corresponding to the rotation speed (usually called superharmonic 1X). In addition, if the system presents a crack, there is a characteristic presence of the components of vibration 1X and 2X at any speed of rotation, corresponding to the speed of rotation and twice the speed of rotation, respectively [49,50]. At rotation speeds close to submultiples of the critical speed $\frac{1}{n}\omega_{c}$, the spectrum is composed of three main superharmonics; the 1X component corresponds to the rotation speed, 2X corresponds to the presence of the crack, and nX corresponds to the critical speed [30,43,47,48].

Regarding the determination of the critical speed of shafts, previous authors have evaluated the critical speed for intact shafts using the workbench of an FEM commercial code [51] as well as experimentally [41]. Gayen et al. [52] obtained the critical speed of a cracked shaft using FEM, considering the effect of the crack by the change in stiffness during the rotation as a cosine function or as an open crack. In [53], a brief review of methods for the approximate measurement of critical speeds in drivelines was presented. However, to the best of our knowledge, the accurate value of the critical speed for a shaft with a breathing crack while in service has not been obtained yet.

In this work, we present a new methodology to determine with precision the critical speed of a cracked shaft, which can be used, for example, in the inverse problem of identification of cracks in rotating shafts. The proposed method uses only the FFT of the transversal displacement of a section of the shaft, and is valid even in cases in which the increments of the displacements are imperceptible. To our knowledge, there is no existing method to calculate the accurate critical speed of a cracked shaft in which the loops of the orbits or the changes of the transverse displacements are not needed.

The rest of this paper is organized as follows. Section 1 is this introduction. In Section 2, we briefly explain the numerical model of the behavior of a cracked shaft with breathing cracks in order to clarify the proposed method. The fundamentals of the model can be found in a previous work ([38]). Section 3 is devoted to explaining the proposed methodology for calculation of the accurate critical speed and its verification. Finally, in Section 4 we show the application of the proposed method to experimental data. We conclude te paper in Section 5 with a summary of our main results.

## 2. Dynamic Behavior of Cracked Shafts

**Figure 1:**
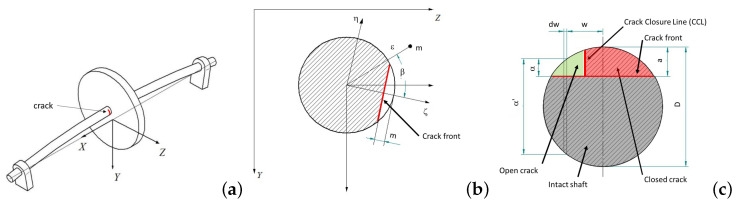
Jeffcott Rotor model: (**a**) schematic configuration, (**b**) fixed and rotary coordinate systems, (**c**) cracked section. Source: Reprinted/adapted with permission from [38]. 2012, Springer Science+Business Media B.V.

The dynamics of a cracked shaft can be analyzed through the widely used Jeffcott Rotor model, which consists of a massless shaft with length *L* and diameter *D* with a crack *a* in its midspan. A disk is attached close to the midspan of the shaft. To complete the Jeffcott Rotor model, an unbalanced eccentricity of mass *m* at a distance ε and orientation β with respect to the crack is considered, along with a a damping coefficient ξ. The details of the cracked Jeffcott Rotor are shown in Figure 1.

The well known equations of motion of a Jeffcott Rotor rotating with an angular speed Ω can be written in the fixed coordinate system Y−Z as

(1) $$\begin{array}{ccc} mY^{\prime \prime }+cY^{\prime }+k_{yy}Y+k_{yz}Z & = & m\varepsilon \Omega^{2}\cos\left(\Omega t+\beta \right)+mg\\ mZ^{\prime \prime }+cZ^{\prime }+k_{zy}Y+k_{zz}Z & = & m\varepsilon \Omega^{2}\sin\left(\Omega t+\beta \right) \end{array}$$

where ${\left(•\right)}^{\prime }$ indicates the time derivative and *c* the damping.

To integrate Equation (1), the stiffness coefficients must be calculated. This has to be done considering the corresponding stiffness coefficients in the rotational frame ζ−η. In order to calculate these, the Stress Energy Release Rate (SERR) and “crack closure line” (CCL) approaches can be used ([28,30,31,37,38]) to consider the closed part of the crack.

The stiffness coefficients can be written in terms of the components of the flexibility matrix as

(2) $$k_{\zeta \zeta }=\frac{g_{\eta \eta }}{g_{\zeta \zeta }g_{\eta \eta }-g_{\zeta \eta }^{2}},\;k_{\eta \eta }=\frac{g_{\zeta \zeta }}{g_{\zeta \zeta }g_{\eta \eta }-g_{\zeta \eta }^{2}},\;k_{\zeta \eta }=k_{\eta \zeta }=\frac{-g_{\zeta \eta }}{g_{\zeta \zeta }g_{\eta \eta }-g_{\zeta \eta }^{2}}$$

where $g_{\eta \eta }$, $g_{\zeta \zeta }$, $g_{\zeta \eta }$, and $g_{\eta \zeta }$ are, according to Fracture Mechanics concepts ([38]),

(3) $$\begin{array}{ccc} g_{\zeta \zeta } & = & \frac{1}{k_{0}}+\int \int \frac{128L^{2}{\hat{\alpha }}^{2}}{E\pi D^{8}}\alpha F_{1}\left(\frac{\alpha }{\hat{\alpha }}\right)d\alpha dw\\ g_{\eta \eta } & = & \frac{1}{k_{0}}+\int \int \frac{512L^{2}w^{2}}{E\pi D^{8}}\alpha F_{2}\left(\frac{\alpha }{\hat{\alpha }}\right)d\alpha dw\\ g_{\zeta \eta } & = & \frac{1}{k_{0}}+\int \int \frac{256L^{2}\hat{\alpha }w}{E\pi D^{8}}\alpha F_{1}\left(\frac{\alpha }{\hat{\alpha }}\right)F_{2}\left(\frac{\alpha }{\hat{\alpha }}\right)d\alpha dw \end{array}$$

where $k_{0}$ is the stiffness of the uncracked rotor, provided by

(4) $$k_{0}=\frac{48L^{3}}{EI}$$

and $F_{1}$ and $F_{2}$ can be written as

(5) $$\begin{array}{ccc} F_{1}\left(\frac{\alpha }{\hat{\alpha }}\right) & = & \sqrt{\frac{2\hat{\alpha }}{\pi \alpha }\tan\left(\frac{\pi \alpha }{2\hat{\alpha }}\right)}\frac{0.923+0.199{\left[1-\sin(\frac{\pi \alpha }{2\hat{\alpha }})\right]}^{4}}{\cos\left(\frac{\pi \alpha }{2\hat{\alpha }}\right)} \end{array}$$

(6) $$\begin{array}{ccc} F_{2}\left(\frac{\alpha }{\hat{\alpha }}\right) & = & \sqrt{\frac{2\hat{\alpha }}{\pi \alpha }\tan\left(\frac{\pi \alpha }{2\hat{\alpha }}\right)}\frac{0.752+2.02\left(\frac{\alpha }{\hat{\alpha }}\right)+0.37{\left[1-\sin(\frac{\pi \alpha }{2\hat{\alpha }})\right]}^{4}}{\cos\left(\frac{\pi \alpha }{2\hat{\alpha }}\right)} \end{array}$$

Finally, the stiffness coefficients in the fixed frame can be obtained from the transformation matrix

(7) $$\left(\begin{array}{cc} k_{yy} & k_{yz}\\ k_{zy} & k_{zz} \end{array}\right)=T^{-1}\left(\begin{array}{cc} k_{\zeta \zeta } & k_{\zeta \eta }\\ k_{\eta \zeta } & k_{\eta \eta } \end{array}\right)T$$

where *T* is the matrix transformation, provided by

(8) $$T=\left(\begin{array}{cc} \cos\Omega t & \sin\Omega t\\ -\sin\Omega t & \cos\Omega t \end{array}\right)$$

### 2.1. Numerical Results: Displacements and Orbits

Here, we consider a shaft of length L=0.9 m and diameter D=0.02 m with a crack at the midspan. The relative depth of the crack $\alpha =\frac{a}{D}$ varies from α=0 to α=0.5. At its midspan, the shaft has a disk made of steel with an eccentric mass. The properties of the eccentric mass are eccentricity ε=0.075 m and angle of eccentricity β=0. The shaft is made of aluminum, with a Young’s Modulus E=72 GPa and Poisson ratio ν=0.3. The damping coefficent is ξ=0.01.

**Figure 2:**
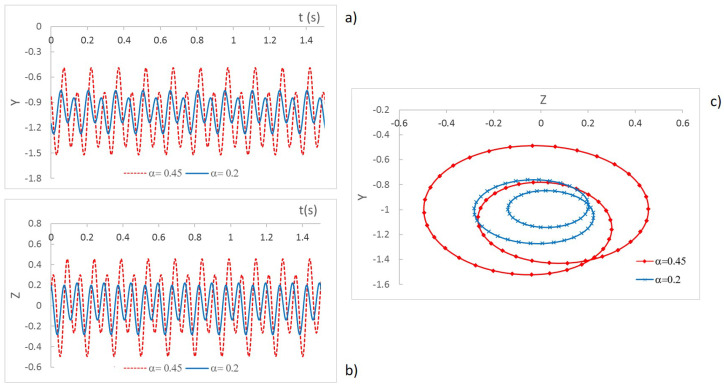
Displacement in mm: (**a**) Y direction, (**b**) Z direction, (**c**) orbits (*p* = 0.495).

The integration of Equation (1) allows the displacements Y−Z of the geometrical center of the shaft located at the cracked section to be obtained for a certain value of the rotation speed Ω. In Figure 2, an example of the displacements and orbits of a shaft with two different cracks, α=0.2 and α=0.45, for a rotation speed p=0.495 can be observed, with *p* being the nondimensional speed and the critical speed of the uncracked shaft $\omega_{nc0}$ provided by

(9) $$p=\frac{\Omega }{\omega_{nc0}}$$

**Figure 3:**
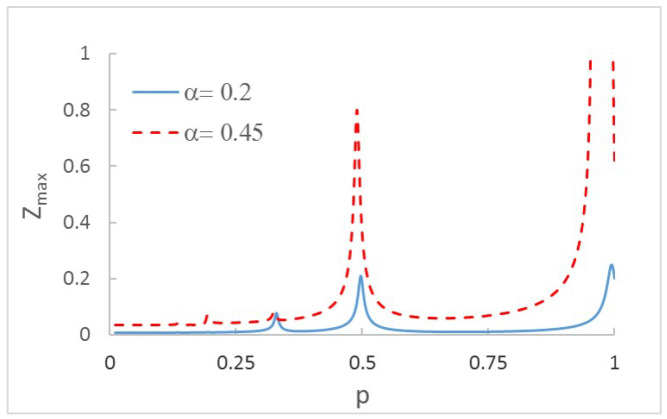
Displacement in the Z direction in mm for the speed rotation sweep and two crack depths: α=0.2 and α=0.45.

The figure shows, as expected, an increment of displacements when the crack is more severe. This increment is even more evident when plotting the orbits. Another interesting aspect to analyze is the increment of the displacements (and orbits) while increasing the rotation speed from p=0 to p=1. In Figure 3, the maximum displacement in direction Z is plotted for for each velocity for two cracked shafts. In this case, two crack depths have been considered: α=0.2 and α=0.45.

As can be seen in the speed sweep, there are a number of speeds at which an increase in displacement is appreciable. Small peaks can be observed around p=0.25 and p=0.33, and bigger peaks at about p=0.50 and p=1.0 These effects, which have previously been obtained by other authors, i.e., [19,20,42,45], appear when the system reaches the proximity of resonance p=1 or the vicinity of other speeds related with resonance, which are called subcritical speeds ($p=\frac{1}{2}$, $p=\frac{1}{3}$, and $p=\frac{1}{4}$). Therefore, when the system reaches speed values close to the critical speed or one of its submultiples, a maximum in the displacements occurs. The amplitude of the peaks depends on the crack size due to the loss of local rigidity (see [20,25]), being greater for more severe cracks, as can be seen in Figure 3.

In order to explain the ongoing results in more detail, an in-depth explanation of Figure 3 is provided. As mentioned before, the natural frequency of a cracked shaft diminishes with the depth of the crack, as does the critical speed. Consequently, the peaks corresponding to a cracked shaft occur at lower speeds than those corresponding to an intact shaft. For a cracked shaft, the deeper the crack, the lower the value of *p* at which the peaks appear. Taking into account that *p* represents the ratio between the rotating speed and the critical speed for a noncracked shaft, the peaks corresponding to the harmonics move to the left as the crack increases in depth. In other words, as the crack depth increases, the critical speed of the shaft decreases.

**Figure 4:**
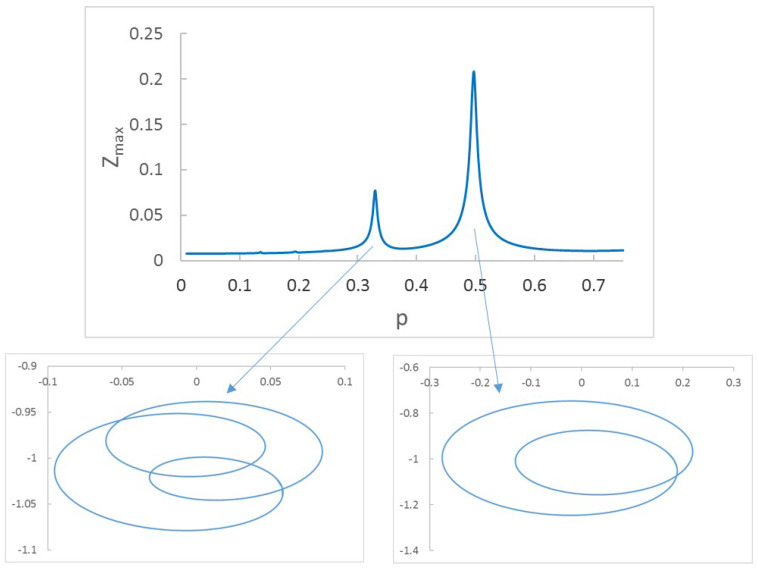
Peaks in sweep and orbits for α=0.2.

Another effect that can be observed during the speed sweep is the change in the shape of the orbits. During the speed sweep, all of the orbits are elliptical except for those at subcritical speeds, for which the elliptical orbits transform into orbits with several inner or outer loops. In Figure 4, the orbits and the sweep up to p=0.75 for a shaft with a crack with depth α=0.2 are shown. It can be observed that there are two main increases in the Z displacement along the sweep at around $p=\frac{1}{3}$ and $p=\frac{1}{2}$. The corresponding orbits have 2 and 1 loops, respectively. It can be said that the subcritical speeds along the sweep can be observed in two ways: first, as abrupt increments of displacements (peaks), and second as the appearance on the loops in the orbits. This leads to a way of identifying the critical speed of the cracked shaft by combining the information provided by the displacements and by the orbits and their loops. If there is a peak at a velocity *p* in which the orbit has *n* loops, it means that $p=\frac{1}{n+1}$ must be one of the subcritical speeds, and thus the critical speed is $p_{c}=\left(n+1\right)p$ ([22,39,40,41,42,43,45]). Although the most direct result (in the absence of loops) would be that obtained for the absolute maximum of the sweep (close to p=1), in most applications the performance of the shafts at that speed can be dangerous, meaning that a relative maxima of the displacement must be chosen instead.

**Figure 5:**
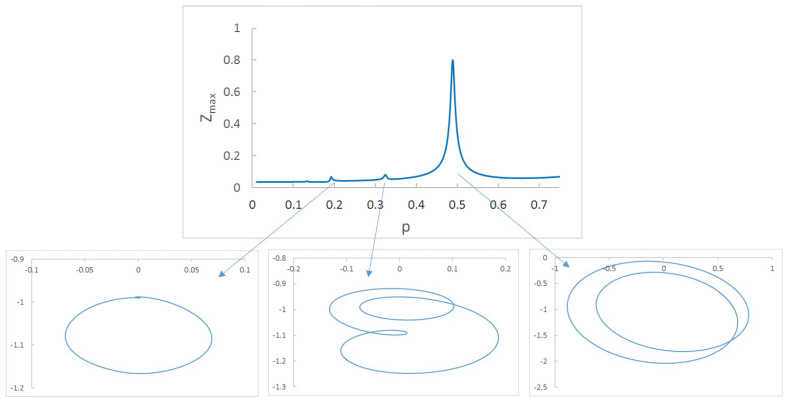
Peaks in sweep and orbits for α=0.45.

Moreover, sometimes the relative maxima are not observable or are not clear enough to identify the value of the corresponding speed. In such cases, this method using the peaks can fail to determine the critical speed of a cracked shaft. This happens mainly when the cracks are very small or when the orbits corresponding to the peaks do not present loops (see Figure 5) [20]. In such cases, the procedure based on the peaks of the rotation speed sweep and the loops in the orbits is not valid for calculation of the critical speed. The problems generated by non-observable data (displacements or orbit loops) can be avoided by using the frequency content of the displacement signal.

### 2.2. Numerical Results: Frequency Content

**Figure 6:**
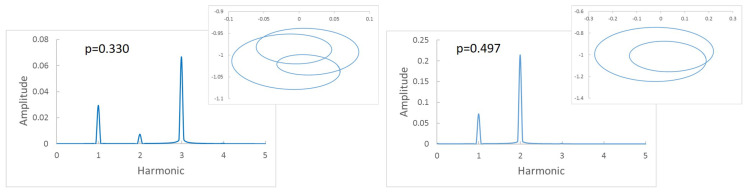
Frequency spectrum of a cracked shaft: α=0.2, p=0.330, and p=0.497.

**Figure 7:**
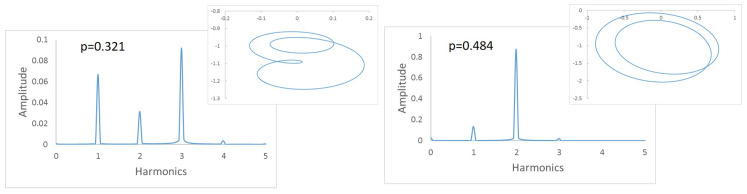
Frequency spectrum of a cracked shaft: α=0.45, p=0.321, and p=0.484.

As mentioned in Section 1, using the Fast Fourier Transform (FFT) with the displacements of the cracked section can provide valuable information on the critical speed of the cracked shaft. In the presence of a crack, for any rotation speed out of the critical or the subcritical ones, the FFT of the displacements provides a spectrum with two harmonics (1X and 2X) corresponding to the rotation speed and twice the rotation speed, although sometimes the second one can be very small. When the rotation speed reaches one of the subcritical speeds, more harmonics are mainly visible: 1X, corresponding to the speed rotation; 2X, corresponding to the presence of the crack; and a third, nX, corresponding to the current subcritical speed. In fact, *n* harmonics appear when the non-dimensional speed of rotation *p* is close to $p=\frac{1}{n}$. The appearance of several harmonics occurs for an interval of speeds around *p* rather than for a single value of *p*. That is, within an interval around *p*, *n* harmonics can be found in every speed of the interval, making the determination of the accurate subcritical speed $p=\frac{1}{n+1}$ impossible, as any of the speeds may correspond to the subcritical speed. Thus, in a practical way, if the frequency spectrum has, for example, three harmonics, two things can be said: first, there is a crack in the shaft (as indicated by the presence of 2X), and second that the current rotation speed is a candidate to be one of the subcritical speeds (as indicated by 1X and 3X). To better illustrate this, a complete example is shown in Figure 6 and Figure 7.

Figure 6a shows the frequency spectrum of a shaft with a crack α=0.2 rotating with a speed p=0.330 and the corresponding orbit. Three peaks and two loops can be observed; thus, that we can say that we are close to $p=\frac{1}{3}$. In Figure 6b, the same shaft is rotating at a speed p=0.497, and there are two peaks and one loop; thus, we can say the rotation speed is around $p=\frac{1}{2}$. The calculated critical speed shows a tiny difference between that obtained at p=0.330 and p=0.497, of $p_{c}=0.990$ and $p_{c}=0.994$, respectively. The same situation is the case for α=0.45 (see Figure 7), where the speeds of rotation are respectively p=0.321 and p=0.484. Here, the critical speed takes the approximate values 0.963 or 0.968. It can be observed that the calculated critical speeds are similar, though not exactly the same, depending on the selected rotation speeds; however, as mentioned before, this value is only an approximate one.

The critical speed determination methods described previously require knowledge of the orbits of the shaft, and consequently, two displacements of the shaft, namely, in both transversal directions, are needed.

## 3. Proposed Method for Determination of the Critical Speed

### 3.1. Determination of the Critical Speed

For many applications, the knowledge of the exact critical speed of a shaft is crucial, for example, with respect to the determination of the presence of a crack in a shaft or the subsequent identification of the crack. The identification procedure is very much related to the natural frequency of the element, which is connected with the critical speed. For this purpose, we are able to use the procedures described in Section 2 to identify the approximate critical speed; however, it is desirable to find the accurate value while using as little data as possible.

**Figure 8:**
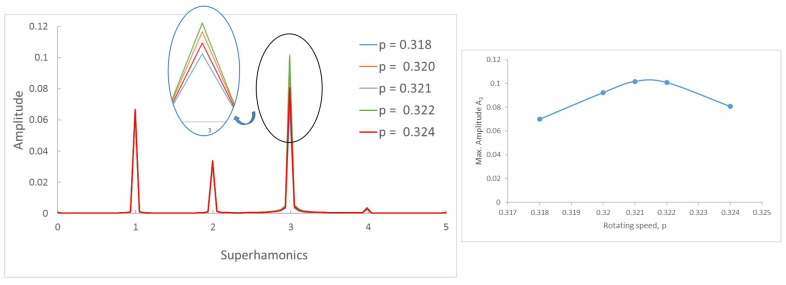
FFT of the transversal displacement for α=0.45 and a speed interval close to $p=\frac{1}{3}$.

**Figure 9:**
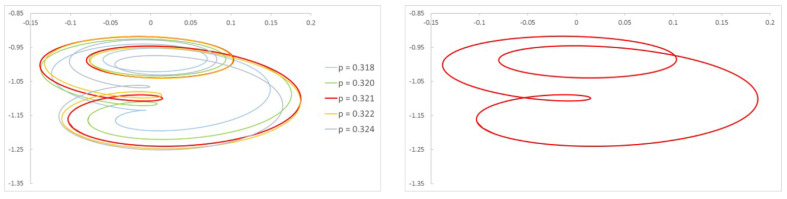
Orbits of the shaft for α=0.45 within a speed interval close to $p=\frac{1}{3}$ and orbit in p=0.321.

To this end, we first investigate how the frequency spectra and the orbits evolve within the interval of speeds in which two or three harmonics (or one or two loops) appear. Following the same example as in the previous Section, in Figure 8 the FFT of one of the displacements in the transversal direction of a cracked shaft (α=0.45) is plotted for speeds within an interval. Here, three peaks are visible in the FFT plot, indicating that the rotation speed is close to $\frac{1}{3}$ of the critical speed. The zoom of the superharmonics 3X, Figure 8a, allows the differences in the amplitude of peak $A_{3}$ to be seen for the analysed rotation speeds. The maximum amplitudes are plotted in Figure 8b, showing their evolution with the speed of rotation *p*. In the analysed case, the highest value of this graph corresponds to a speed of p=0.321. Consequently, the critical speed is $p_{c}=3\times p=0.962$. Figure 9 shows the view of the orbits corresponding to the different speeds of rotation within the same interval. The three loops and the speed corresponding to the largest orbit due to resonance allows us to reinforce the previous result for the critical speed, although the orbit representation is not needed to determine the critical speed.

**Figure 10:**
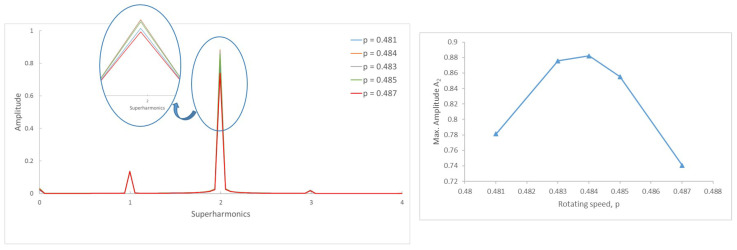
FFT of the transversal displacement for α=0.45 and a speed interval close to $p=\frac{1}{2}$.

**Figure 11:**
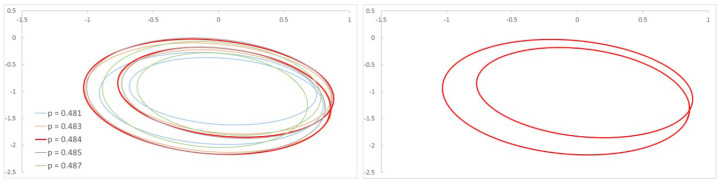
Orbits of the shaft for α=0.45 within a speed interval close to $p=\frac{1}{2}$ and orbit in p=0.484.

The same situation is reflected in Figure 10. Here, the two peaks in the FFT lead to the conclusion that the subcritical speed is within the interval around $p=\frac{1}{2}$. The maximum of the 2X harmonic corresponds to p=0.484, meaning that the exact critical speed of this cracked shaft is $p_{c}=2\times p=0.968$. Furthermore, the single loop in Figure 11 confirms that the speed is close to $p=\frac{1}{2}$. The largest orbit additionally confirms that the critical speed is p=0.968.

The critical speeds obtained with the data at $p=\frac{1}{3}$ and $p=\frac{1}{2}$ are very similar, at p=0.962 and p=0.968, the same as for the previous methods.

**Figure 12:**
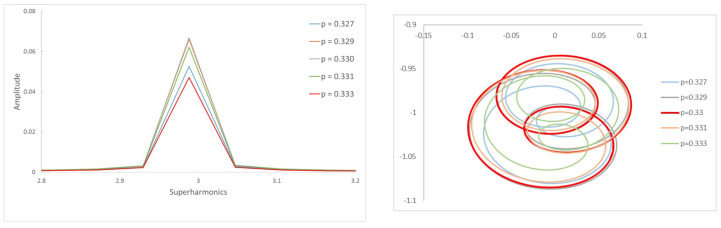
FFT of the transversal displacement and orbits for α=0.2 and a speed interval close to $p=\frac{1}{3}$.

**Figure 13:**
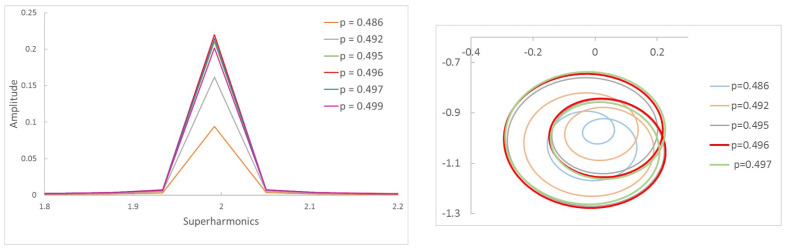
FFT of the transversal displacement and orbits for α=0.2 and a speed interval close to $p=\frac{1}{2}$.

This procedure provides results with the same accuracy for other crack depths. In order to illustrate this, in Figure 12 and Figure 13 we show the harmonics 3X and 2X of the FFT for a shallow crack (α=0.2), providing further reinforcement. The obtained critical speeds for this case are $p_{c}=0.990$ and $p_{c}=0.992$, respectively.

The proposed methodology can be summarized as follows (see, for example [54]). Depending on the harmonics present in the frequency sprectrum, an ideal unbalanced shaft such as that analyzed theoretically and with no other defects (i.e., no clearances or misaligments) offers several situations:

- only 1X indicates an intact unbalanced shaft.
- only 1X and 2X indicate the presence of a crack. The current speed may or may not be the subcritical speed $p=\frac{1}{2}$, as the two harmonics can indicate other kinds of situations that are not cracks.
- 1X, 2X, ...nX indicates the presence of a crack and a subcritical speed $p=\frac{1}{n}$

In the case of a real cracked shaft (with clearances and misaligments included), the above situation changes as follows:

- 1X and other harmonics 2X, ...nX, while very small with respect to 1X, indicate an intact shaft with other possible defects (i.e., clearances).
- 1X and 2X and other small harmonics indicate a defect (i.e., cracks or clearances). The speed may or may not be the subcritical speed $p=\frac{1}{2}$, for the same reasons as before.
- 1X, 2X, ...nX with 1X, 2X, and nX larger than the other harmonics indicates the presence of a crack and a subcritical speed of $p=\frac{1}{n}$
- 1X, 2X, ...nX with 1X and nX larger than the other harmonics indicates an intact shaft and a subcritical speed of $p=\frac{1}{n}$

### 3.2. Verification of the Method

**Figure 14:**
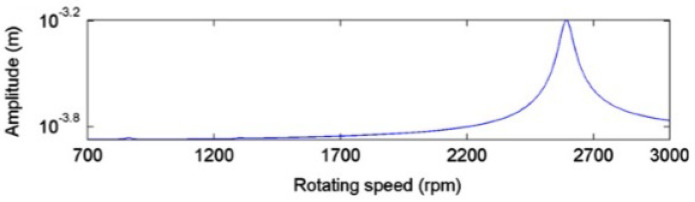
Amplitude of signal of the displacement at speed sweep for α=0.01. Reprinted/adapted with permission from [45]. 2013. Elsevier.

To verify the proposed method for calculating the critical speed, we applied it to results from the literature. In particular, we analyzed the results of the transversal displacements provided by Guo et al. [45]. Figure 14 shows the amplitude of the displacement obtained by Guo et al. for a cracked shaft with a crack of depth α=0.01. An increase in displacement at speeds between 2200 rpm and 2700 rpm can be observed. Guo et al. indicate that the critical speed corresponding to this cracked shaft is 2592 rpm.

**Figure 15:**
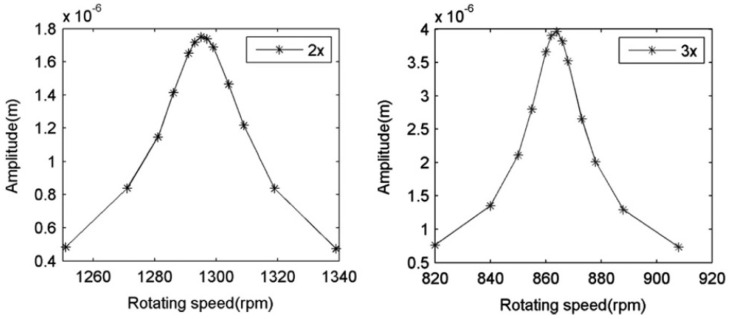
Amplitudes of 2X and 3X at speeds close to $p=\frac{1}{2}$ and $p=\frac{1}{3}$ for α=0.01. Reprinted/adapted with permission from [45]. 2013. Elsevier.

In their article, Guo et al. show results for the 2X and 3X superharmonic components obtained from the original displacement signal by means of the EMD method for decomposition into several separate IMFs at speeds close to $p=\frac{1}{2}$ and $p=\frac{1}{3}$ (Figure 15).

Table 1 shows the results of the maximum value of the amplitudes of the second harmonic 2X obtained in [45] along the speed interval around $p=\frac{1}{2}$. In this case, the maximum is reached at Ω=1295.29 rpm. Thus, according to the proposed method, this is the value corresponding to $p=\frac{1}{2}$, and hence the critical speed is $\omega_{c}=p\times 2=2590.58$ rpm, which is very close to that indicated by Guo et al.

Table 2 shows the results obtained by [45] for the maximum of the third harmonic, 3X, corresponding to the speed range of $p=\frac{1}{3}$, for the same crack depth. In this case, the maximum is reached at Ω=864.28 rpm. Using the proposed procedure, the value corresponds to $p=\frac{1}{3}$; hence, the critical speed is $\omega_{c}=2592.84$ rpm, a value even closer to that provided by Guo et al. Therefore, the proposed method of calculating the critical speed from the experimental data is verified with an error of $10^{-4}$.

## 4. Experimental Results

After validation, the proposed methodology was applied to the experimental results of a set of rotating cracked shafts.

### 4.1. Experimental Setup

**Figure 16:**
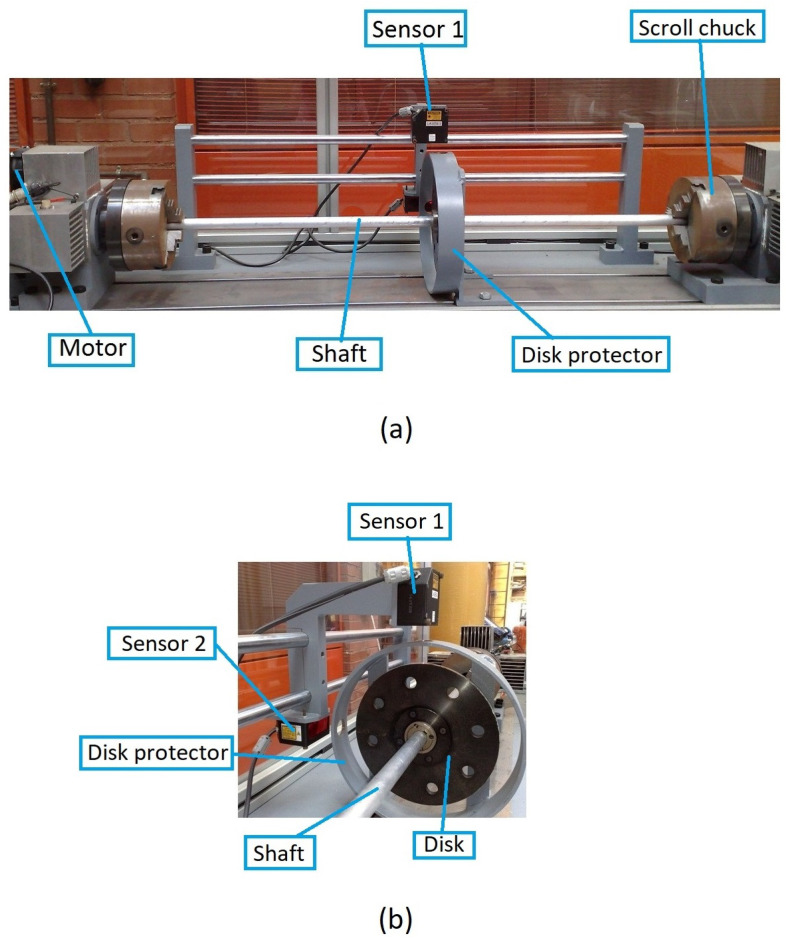
(**a**) Layout of the shaft and (**b**) disk arrangement.

The layout of the shaft to be tested is similar to that known in the literature as a Jeffcott Rotor. This consists of a shaft having a disk at the midspan, as shown in Figure 16a. The bench has a drag head driven by a servomotor, which permits testing shafts of different lengths and diameters as well as varying the speed of rotation.

The disk arrangement can be seen in Figure 16b. The disk has a series of holes, which allows coupling of one or more eccentric masses to produce an imbalance during the test. The bench includes laser sensors (OMRON LS-LD50) with an accuracy of 0.25 μm and a sampling frequency of 10 kHz for measurement of the horizontal and vertical displacements.

**Figure 17:**
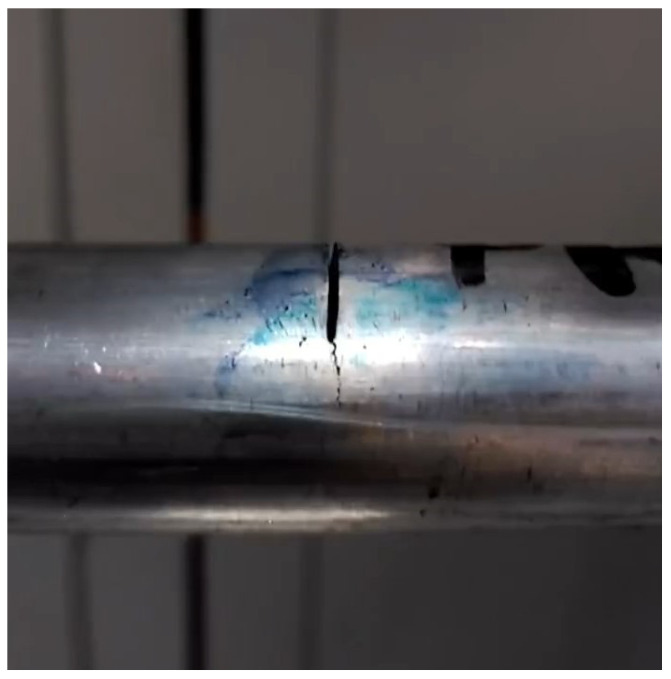
Detail of the notch and the crack of one of the shafts.

In the present work, tests were carried out on shafts at variable rotation speeds from p=0 to p=0.687, as it was necessary to carry out the experiment away from the resonance value for safety reasons. The speed of rotation was non-dimensionalized with the critical speed of the uncracked shaft. For this study, a set of cracked shafts with a length of L=0.900 m and a diameter of D=0.020 m was tested. The tested shafts were made of aluminum, with a Youngś Modulus E=72 GPa, Poisson ratio ν=0.33, and density ρ = 2700 kg/m${}^{3}$. The disk located at the midspan had a diameter d=0.200 m, thickness $e_{d}=0.021$ m, and mass $m_{d}=5$ kg. This disk is made of steel with Youngś Modulus E=210 GPa, density ρ = 7850 kg/m${}^{3}$ and Poisson ratio ν=0.3. The eccentric mass, which was placed in one of the holes of the disk, had a value $m_{e}=0.2$ kg, and was located at a distance e=0.075 m from the center of the disk. The eccentricity orientation in relation to the crack was taken as β=0 rad. The cracks of the shafts had a relative depth α=0.1, 0.15, 0.2, 0.25, 0.3, 0.35, 0.4. A detailed image of the crack with a previous notch of one of the shafts can be seen in Figure 17

### 4.2. Experimental Application of the Method and Results

**Figure 18:**
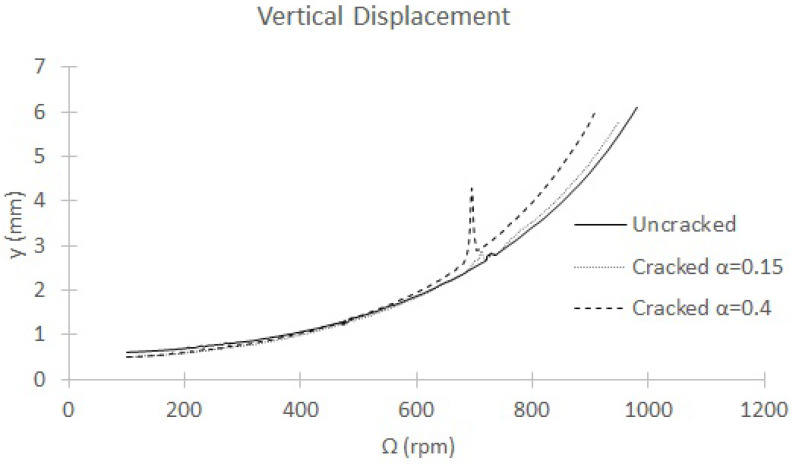
Vertical displacement in the speed sweep.

The data obtained from the experimental tests are the horizontal and vertical displacements of the central section in a speed sweep. In this work, only vertical displacements data are taken at every $p=7e^{-4}$. In Figure 18, the maximum amplitude of the vertical displacement in each rotation speed for an uncracked shaft and two cracked shafts (α=0.15 and α=0.4) is shown. An abrupt increase in displacement at about Ω=700 rpm can be observed for the shaft with a deep crack (α=0.4). However, the increase in the displacement is negligible for the uncracked shaft and for the shaft with a shallow crack (α=0.15). In the cases analyzed in this paper, shown in Figure 18, the rotation speed at which an increment in the displacement is observed varies from approximately Ω=730 rpm for the uncracked shaft to approximately Ω=690 rpm for the cracked shaft with α=0.4. This can be used as an indicator of the presence of the crack as well as a procedure to determine the critical speed of the shaft.

The increment of displacements observed around certain rotation speeds do not provide enough information on their own to determine the critical speed due to the difficulty of determining the exact value. For this reason, data processing is necessary. The number of inner loops that appear in the orbits and the frequency spectrum analysis (FFT) have been previously used by other authors [30,42,43,45,46,47,48], as mentioned earlier in this paper. When the increase in displacement is appreciable at a rotation speed *p*, for example in the case of large crack sizes such as α=0.4 in Figure 18, the orbit loops (if they are observable) or the number of the harmonics (which are always perceptible) allows the determination of the submultiple of the critical speed, and thus the critical speed itself, in an accurate way. However, when the increments in the displacement are not appreciable, for example, in the case of small crack sizes around α=0.15 and for uncracked shafts, such as those in Figure 18, the orbit loops and the harmonics in the frequency spectrum are useful for determining the rotation speed interval of the submultiple of the critical speed. However, at times even the orbits do not offer enough information. For this reason, in this work we use the method based on only the frequency content provided by the FFT of one of the transversal displacements.

**Figure 19:**
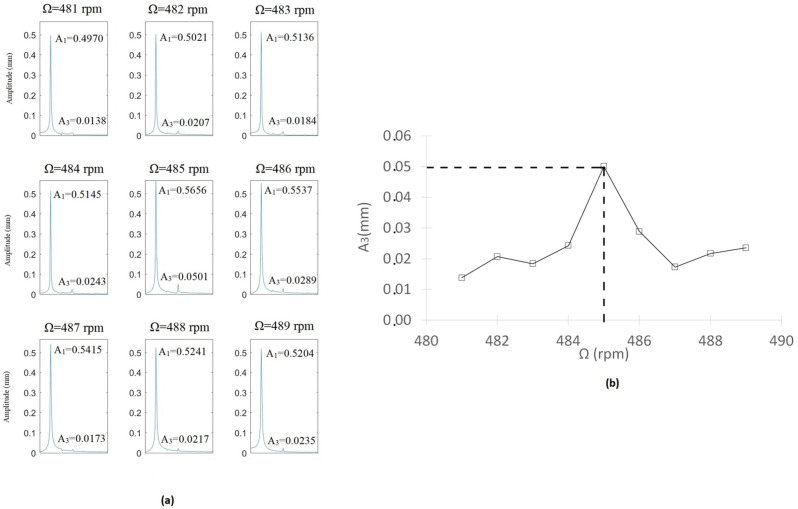
FFT of the displacements of the intactshaft around 3X. (**a**) FFT for different speeds of rotation. (**b**) Maximum values of the amplitude of $A_{3}$ in each speed.

The first test we carried out was that corresponding to the uncracked shaft, with the aim of obtaining the critical speed of the intact shaft and non-dimensionalizing the speeds of the cracked shafts. Following the approach indicated in the previous section, the evaluation is carried out where the third harmonic appears. Looking to the surrounding speeds at which 3X exists, the maximum amplitude of the third harmonic provides the critical speed of the uncracked shaft, as explained before. In the present case (Figure 19), the maximum value of 3X corresponds to Ω=485 rpm, which provides the critical speed of the intact shaf as $\Omega_{nc0}=1455$ rpm. The obtained results when following the same procedure while taking into account the other subcritical speed ranges are presented in Table 3.

There are differences in the values obtained for the critical speeds using the three subcritical speeds and the two displacements. One of the critical speeds, that obtained with the $p=\frac{1}{3}$ and vertical displacement, is used for non-dimensionalizing the critical speeds of the cracked shafts in order to compare them. The selection of the critical speed from among these is explained after the whole experimental analysis.

After the critical speed of the uncracked shaft is obtained, the critical speeds of other shafts with the same geometrical and material properties and cracks of different sizes can be obtained as follows.

**Figure 20:**
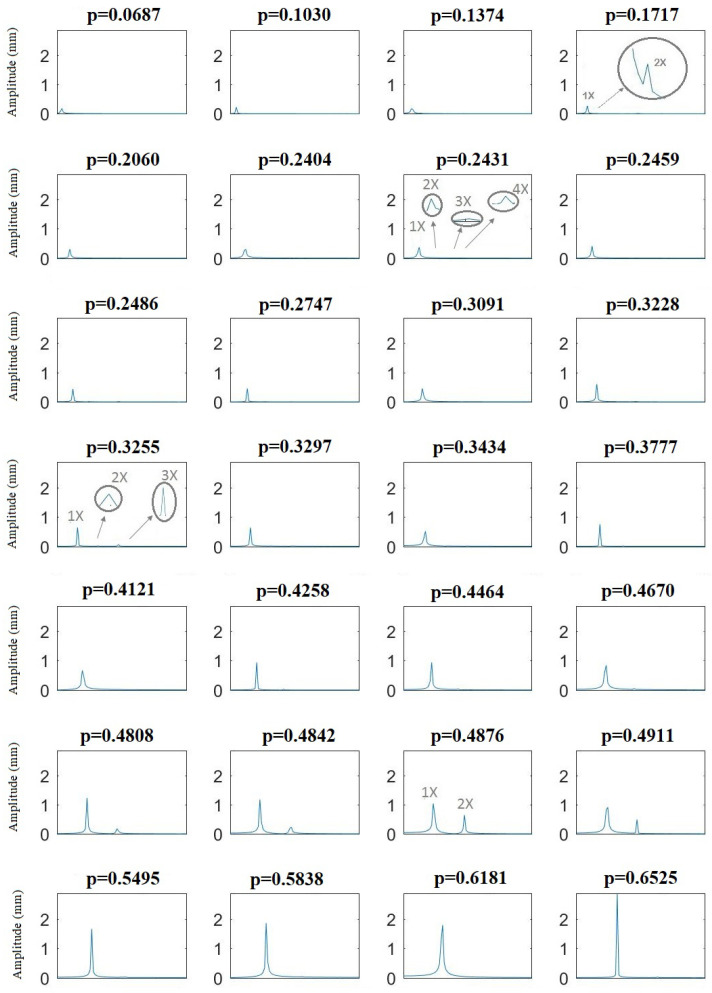
FFT of the vertical displacement at different rotation speeds for the case of α=0.15.

The FFT is applied to the vertical displacements in all cases. In Figure 20, the frequency contents of a cracked shaft with a crack depth of α=0.15 along the speed sweep from p=0.0687 to p=0.6525 are shown. Each image corresponds to the FFT of the vertical displacements in a particular speed of rotation. Here, the speed of rotation has been non-dimensionalized using the critical speed of the uncracked shaft. Although it is sometimes difficult to identify due to the scale, there are four harmonics at speeds near p=0.25, three harmonics at speeds around p=0.33, and two harmonics at speeds near p=0.5.

**Figure 21:**
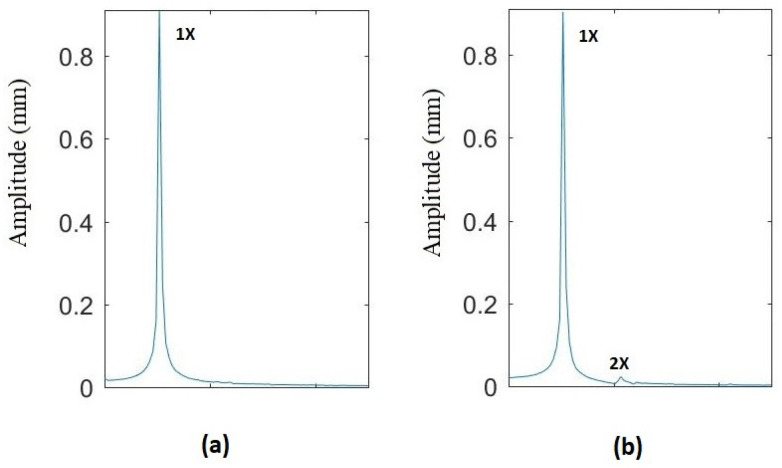
FFT at p=0.433: (**a**) uncracked shaft and (**b**) cracked shaft α=0.15.

**Figure 22:**
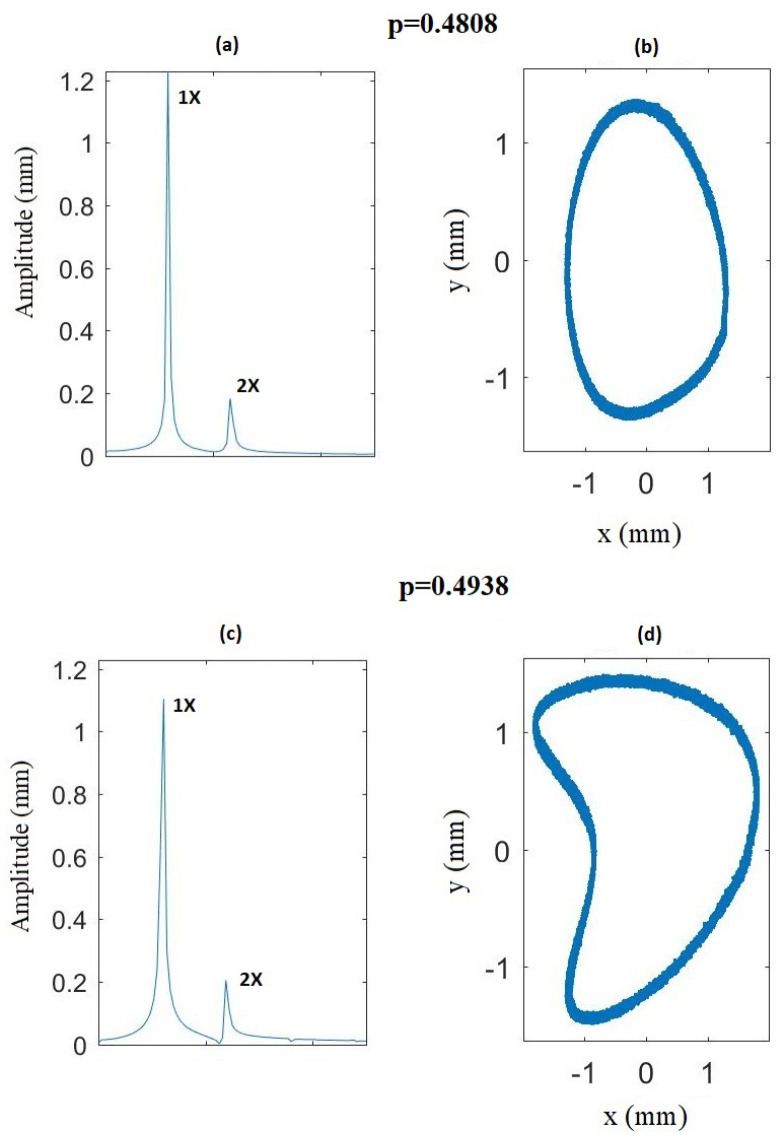
Comparison of the results at two different rotation speeds for α=0.15: (**a**) FFT at p=0.4808, (**b**) orbit at p=0.4808, (**c**) FFT at p=0.4938 (**d**) orbit at p=0.4938.

Even though in most of the images only the 1X harmonic is observable due to the scale, at least two vibration components (1X and 2X) appear in the frequency spectrum at any rotation speed. This can be best seen in Figure 21, where the FFT for (a) an uncracked shaft and (b) a cracked shaft (α=0.15) are shown for a rotation speed p=0.433 (which does not correspond to any harmonic). In the case of the uncracked shaft (Figure 21a), only one harmonic (1X, which corresponds to the rotating speed) appears. In the case of the cracked shaft (Figure 21b), two harmonics, 1X and 2X, appear, corresponding to the rotation speed and twice the rotation speed, respectively, indicating the presence of the crack. As 2X appears at any rotation speed in the presence of a crack, it is impossible to determine the critical speed using this range of speeds. In this situation, the shape of the orbits must be analyzed in order to determine whether or not the rotation speed is close to $p=\frac{1}{2}$. In Figure 22, the FFT (a and c) and the orbits (b and d) of the two different rotation speeds, p=0.4808 and p=0.4938, are represented for the case of α=0.15. In both cases, two harmonics appear in the FFT; thus, that the orbit shape is analyzed to determine the subcritical speeds interval. It can be seen that there is an elliptical orbit for p=0.4808 and a non-elliptical one (assuming the beginning of a loop) for p=0.4938. Therefore, p=0.4938 is within the subcritical speed interval of $p=\frac{1}{2}$, and p=0.4808 is not.

To avoid the use of the orbits, i.e., in the case in which only the displacement in one direction is provided, the proposed methodology allows the exact submultiple of the critical speed to be identified using speed ranges other than $p=\frac{1}{2}$. For this, a detailed sweep around other subcritical speeds needs to be made.

**Figure 23:**
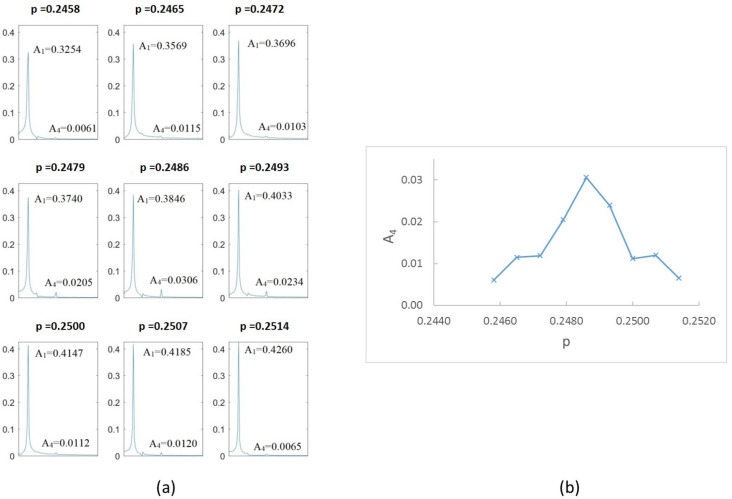
(**a**) FFT for α=0.15 at speeds close to $\frac{1}{4}\Omega_{c}$ and (**b**) amplitude $A_{4}$ versus rotation speed for α=0.15.

**Figure 24:**
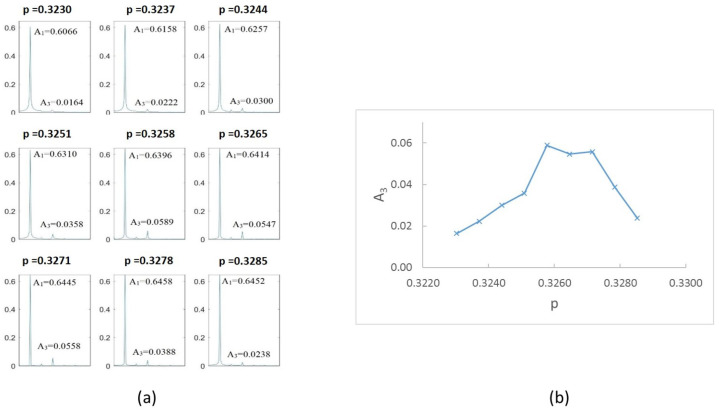
(**a**) FFT for the case α=0.15 at speeds close to $\frac{1}{3}\Omega_{c}$. (**b**) Amplituds $A_{3}$ versus rotation speed for α=0.15.

**Figure 25:**
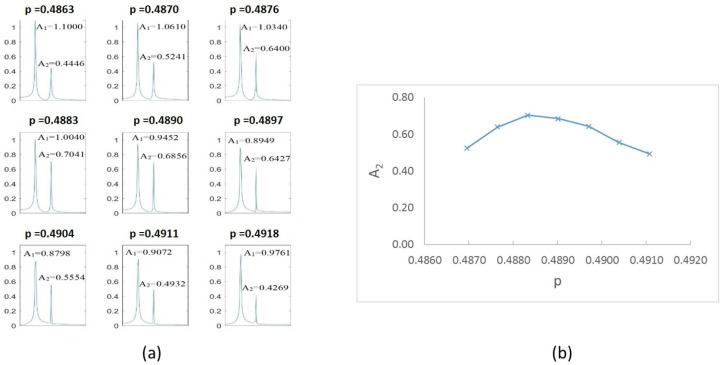
(**a**) FFT for α=0.15 at speeds close to $\frac{1}{2}\Omega_{c}$ and (**b**) amplitude $A_{2}$ versus rotation speed for α=0.15.

Figure 23a shows the FFT obtained for different speeds within the interval close to $p=\frac{1}{4}$, that is, from p=0.242 to p=0.247. The most representative results are shown, and the values of the amplitudes 1X and 4X are indicated. In Figure 23b, the value of the superharmonic 4X, $A_{4}$ is represented against the rotation speed. The maximum value along the speed sweep corresponds to p=0.244; according to the proposed method, this speed is an accurate submultiple of the critical speed. The same representation can be made for the speed ranges of $p=\frac{1}{3}$ and $p=\frac{1}{2}$; see Figure 24 and Figure 25. The most representative results are shown, and the values of the amplitudes 3X and 2X are indicated. The maximum value along the speed sweeps corresponds to p=0.326 and p=0.488, with these speeds being accurate submultiples of the critical speed in the corresponding intervals.

The critical speed can be calculated using Equation (9) to obtain $p_{c}=0.976$ using the 4X and 2X harmonics and to obtain $p_{c}=0.978$ using the 3X harmonic, with only a very tiny difference among them due to the resolution of the equipment.

**Figure 26:**
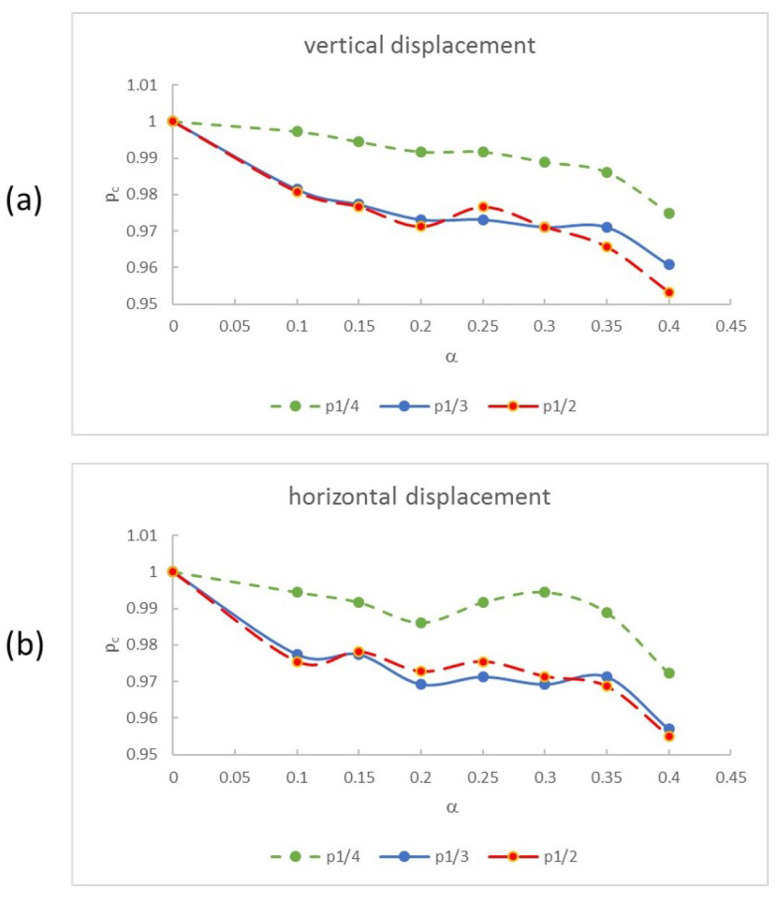
Critical speeds for $p_{c}$ and crack depth α: (**a**) when using vertical displacements and (**b**) when using horizontal displacements.

The results for the same operation carried out on the other shafts with increasing crack depths are shown in Figure 26, where the calculated critical speeds are represented for each crack depth for both the vertical and the horizontal displacements. Note that the critical speed decreases with the increment of the severity of the crack, following the tendency of the natural frequency with respect to crack growth.

Regarding the speed range in which the critical speed is calculated, there are two ranges, $p=\frac{1}{3}$ and $p=\frac{1}{2}$, that offer very similar results no matter the depth of the crack. In general, there is very good agreement between the results for speeds $p=\frac{1}{3}$ and $p=\frac{1}{2}$, and any of them can be chosen for calculating the critical speed of the cracked shaft. On the contrary, in the range $p=\frac{1}{4}$ an over-estimation of the critical speed is obtained. Because calculation of the critical speed of the intact shaft requires $p=\frac{1}{3}$, better than $p=\frac{1}{2}$, and taking into account that the results are very similar for $\frac{1}{3}$ and $\frac{1}{2}$ and very different for $\frac{1}{4}$, in the case of cracked shafts the choice of how to determine the critical speed of the intact shaft corresponds to the range $p=\frac{1}{3}$. Regarding the displacement used to obtain the frequency content and the harmonics (see Figure 26), the calculation of the critical speed offers results with the same accuracy whether using vertical or horizontal displacements, and they can be used indiscriminately.

## 5. Conclusions

In this paper, we propose a method for determining the accurate critical speed of a cracked shaft. The proposed method is explained theoretically using the results obtained by solving the equations of motion for a cracked shaft, then verified using data from the literature. The method is applied to the experimental data of an unbalanced cracked shaft with increasing crack depth, including an intact shaft.

The proposed methodology allows the presence of a crack to be identified by analyzing the harmonics of the frequency spectrum of the transversal displacements of the shaft at the subcritical speeds of rotation. When a crack is identified, the critical speed is calculated using the amplitudes of the harmonics. The proposed procedure allows the critical speed of any cracked shaft to be calculated, regardless of crack size.

Our experimental results corroborate the assumption that the presence of a crack diminishes the critical speed, with the speed dropping as the crack becomes more severe. The best results are achieved when the displacements are analyzed at speeds within the range of $\frac{1}{2}$ or $\frac{1}{3}$ of the critical speed. Moreover, the best results are obtained irrespective of whether the vertical or the horizontal displacements are used. For practical applications, the critical speed must be calculated at a rotation speed within the range of $p=\frac{1}{3}$ or at a speed at which three harmonics appear, as two harmonics are not certain to be within the second subcritical speed.

A highlight of the proposed procedure is that knowledge of the critical speed of the intact shaft is not necessary (although it can be obtained), as the number and amplitude of the harmonics are sufficient to detect both the crack and the value of the critical speed. Another important key element of the proposed procedure is that knowledge of the orbits is not necessary; only one displacement measurement, and consequently only one measurement device, is needed to calculate the critical speed, instead of the two measurements required by other approaches for the same purpose.

## Figures and Tables

**Table 1:** Maximum amplitudes of the IMF at speeds close to $p=\frac{1}{2}$ for α=0.01 in [45].

Ω (rpm)	Amplitude of 2*X*
1271.18	0.84 × $10^{-6}$
1281.18	1.17 × $10^{-6}$
1286.47	1.43 × $10^{-6}$
1291.17	1.76 × $10^{-6}$
1295.29	1.79 × $10^{-6}$
1297.06	1.77 × $10^{-6}$
1299.41	1.72 × $10^{-6}$
1304.71	1.48 × $10^{-6}$
1309.41	1.24 × $10^{-6}$

**Table 2:** Maximum amplitudes of the IMF at speeds close to $p=\frac{1}{3}$ for α=0.01 in [45].

Ω (rpm)	Amplitude of 3*X*
850.15	2.08 × $10^{-6}$
855.06	2.75 × $10^{-6}$
859.98	3.63 × $10^{-6}$
861.82	3.83 × $10^{-6}$
864.28	3.90 × $10^{-6}$
866.13	3.75 × $10^{-6}$
867.97	3.48 × $10^{-6}$
872.89	2.60 × $10^{-6}$
878.43	1.98 × $10^{-6}$

**Table 3:** Critical speeds for the uncracked shaft obtained at different rotation speeds.

From	$\Omega_{nc\frac{1}{4}}$ rpm	$\Omega_{nc\frac{1}{3}}$ rpm	$\Omega_{nc\frac{1}{2}}$ rpm
Horizontal displacement	1440	1464	1470
Vertical displacement	1432	1455	1456

## Data Availability

Not applicable.
